# Novel compound variants of the *AR* and *MAP3K1* genes are related to the clinical heterogeneity of androgen insensitivity syndrome

**DOI:** 10.1042/BSR20200616

**Published:** 2020-05-07

**Authors:** Yiping Cheng, Yan Sun, Yiming Ji, Dongqing Jiang, Guoxin Teng, Xiaoming Zhou, Xinli Zhou, Guimei Li, Chao Xu

**Affiliations:** 1 Department of Endocrinology and Metabolism, Shandong Provincial Hospital Affiliated to Shandong University, 324, Jing 5 Road, Jinan 250021, Shandong, China; 2 Department of Endocrinology and Metabolism, Shandong Provincial Hospital Affiliated to Shandong First Medical University, 324, Jing 5 Road, Jinan 250021, Shandong, China; 3 Institute of Endocrinology, Shandong Academy of Clinical Medicine, Jinan 250021, Shandong, China; 4 Shandong Clinical Medical Center of Endocrinology and Metabolism, Jinan 250021, Shandong, China; 5 Department of Pediatrics, Shandong Provincial Hospital Affiliated to Shandong University, 324, Jing 5 Road, Jinan 250021, Shandong, China; 6 Department of Endocrinology and Metabolism, the Second Hospital of Shandong University, Jinan 250033, Shandong, China; 7 Department of Pathology, the Second Hospital of Shandong University, Jinan 250033, Shandong, China

**Keywords:** androgen-insensitivity syndrome, diagnosis, disorders of sex development, genetics

## Abstract

Androgen insensitivity syndrome (AIS; OMIM 300068) is the most frequent cause of 46, XY disorders of sex development (DSD). However, the correlation between genotype and phenotype has not been determined. We conducted a systematic analysis of the clinical characteristics, hormone levels, ultrasonography data and histopathology of a 46, XY Chinese patient with AIS. The family was followed up for nearly 8 years. We applied whole-exome sequencing (WES) for genetic analysis of the pedigree and performed bioinformatic analysis of the identified variants. Human embryonic kidney 293T/17 (HEK293T/17) cells were transiently transfected with wild-type or mutant *AR* and *MAP3K1* plasmid. Cell lysates were used to analyze androgen receptor (AR) production. A novel hemizygous *AR* variant (c.2070C>A, p. His690Glu) and a rare heterozygous *MAP3K1* variant (c.778C>T, p. Arg260Cys) were identified by WES in the proband and her mother. Bioinformatic analysis predicted these two variants to be pathogenic. Multiple amino acid sequence alignments showed that p. His690 and p. Arg260 are conserved among various species. His690Glu is a mutation that decreased the AR production, whereas the Arg260Cys mutation increased the AR production. The novel compound variants of the *AR* and *MAP3K1* genes also increased the production of AR protein. Thus, the phenotype of the patient may be caused by defects in both the *AR* and *MAP3K1* signaling pathways. Compound variants of the *AR* and *MAP3K1* genes resulted in a specific phenotype in this patient with AIS. WES might reveal genetic variants that explain the heterogeneity of AIS.

## Introduction

Androgen insensitivity syndrome (AIS; OMIM 300068) is the most frequent cause of 46, XY disorders of sex development (DSD). AIS is characterized by a patient exhibiting a 46, XY karyotype and a normal amount of testosterone secretion but with symptoms and signs of androgen deficiency or reduction, such as female external genitalia, blind vagina, and uterine absence [1,2]. It is estimated that the prevalence of complete AIS is 1 in every 40,000–60,000 births, though the prevalence of partial AIS is unknown [3]. AIS is inherited in an X-linked recessive manner and is caused by variants in the *androgen receptor* (*AR*) gene [4], which is located on chromosome Xq11-12, that consists of 8 exons and encodes an androgen receptor containing 920 amino acids [5]. More than 1000 different *AR* gene variants have been identified in AIS patients [6]. However, the correlation between genotype and phenotype has not yet been determined [7]. The same *AR* variant may cause significant variation in the degree of masculinization in different individuals, even among members of the same family. Furthermore, the exact cause of this variation is not well understood. With the development of next-generation sequencing, useful information for explaining this heterogeneity, such as unidentified coactivator proteins, is expected to be forthcoming.

AIS exhibits overlapping clinical phenotypes with other DSDs, such as ovotesticular DSD and 5α-reductase 2 deficiency [8,9]. Müllerian derivatives are generally not found in individuals with AIS. Moreover, few cases of sporadic AIS have been reported due to the appearance of incomplete Müllerian regression [10–12]. In addition to the *AR* gene, there are many other genes that cause disorders of sex development, such as the *MAP3K1* gene. *MAP3K1* variants were first described in two large families and two of 11 sporadic cases of 46, XY DSD in 2010 [13]. Recent studies have shown that *MAP3K1* is one of the more common genes mutated in 46, XY DSD, with 13–18% of these patients showing variants of this gene [14]. The *MAP3K1* gene is located at 5q11.2 and encodes a key serine/threonine protein kinase (MAP3K1) of approximately 196 kD in the MAPK pathway that activates the extracellular signal-regulated kinase (ERK) and c-Jun NH2-terminal kinase (JNK) pathways and plays an important role in cell proliferation, differentiation, and apoptosis [15]. As both *MAP3K1* and *AR* variants can cause 46, XY DSD, the question of whether a correlation exists between the two molecules needs to be addressed.

Interestingly, we detected a compound variant of *MAP3K1* and *AR* in a 46, XY Chinese patient with AIS. The patient presented with bilateral inguinal masses (pathologically confirmed as bilateral testicular tissue), female external genitalia (underdeveloped labia and clitoris), and the presence of a uterus, which was indicative of incomplete Müllerian regression and did not match the typical characteristics of AIS. A novel hemizygous *AR* variant (c.2070C>A, p. His690Glu) and a rare heterozygous *MAP3K1* variant (c.778C>T, p. Arg260Cys) were identified by whole-exome sequencing (WES) in the proband and her mother. Bioinformatic analysis and *in vitro* experiments showed that these two variants are pathogenic. Our study emphasizes the heterogeneity of AIS, which has significance for research on the heterogeneity of diseases.

## Subjects and methods

### Subjects

The Chinese patient with AIS in our study, in whom bilateral inguinal hernias were discovered, was 2.6 years of age in November 2011. We collected her clinical characteristics, physical examination results, hormone levels, ultrasonography data, and pathological examination results to determine the location and nature of the gonads and the appropriate surgical intervention. Peripheral blood samples were obtained from the patient and her parents.

### Follow-up studies

We followed this family for nearly 8 years. We closely tracked the patient’s urogenital development, intellectual development, drug treatment regimens and potential complications, and developments such as psychological and cognitive impairment, gender anxiety disorder, and the presence of gonadal tumors.

### Whole-exome sequencing and Sanger sequencing

Genomic DNA was isolated from peripheral blood leukocytes using QIAamp DNA Mini Kit (Qiagen, Germany) following the manufacturer’s instructions. WES was performed using DNA from peripheral blood. After genomic DNA fragmentation, paired-end adaptor ligation, amplification and purification, human exons were captured by using SeqCap EZ Med Exome Enrichment Kit (Roche NimbleGen, U.S.A.). A DNA library was generated by postcapture amplification and purification and then sequenced with the Illumina HiSeq sequencing platform. Sequence data alignment to the human genome reference (hg19) and variant calling were performed with NextGene V2.3.4 software to obtain the coverage and mean read depth of target regions. The average coverage of the exome was >100×, which permitted examination of the target region with sufficient depth to exactly match >99% of the target exome. To ensure the accuracy of data analysis, mutations with low coverage in the target area were filtered.

Additionally, annotation information, including the conservation of nucleotide bases and amino acids, predictions of biological functions, frequency in normal populations (Genome Aggregation Database (GnomAD), Trans-Omics for Precision Medicine (TOPMED), Exome Aggregation Consortium (ExAC), and data from Human Gene Mutation Database (HGMD), Clinvar and Online Mendelian Inheritance in Man (OMIM) databases, was performed by using NextGene V2.3.4 and our in-house scripts. A variant was recognized as a mutation when it was not found in dbSNP (http://www.ncbi.nlm.nih.gov/snp/), in the exome variant server (http://evs.gs.washington.edu/EVS/), in the ensemble database and in 500 Chinese controls or when the allele frequency was found to be <0.001 in the database. Pathogenic variants were determined according to Standards and Guidelines for the Interpretation of Sequence Variants published by the American College of Medical Genetics and Genomics (ACMG) in 2015 with Human Genome Variation Society (HGVS) nomenclature.

We used WES to detect candidate variants. Detected pathogenic or suspected pathogenic variants were verified by Sanger sequencing, ensuring that the coverage of the gene coding sequence reached 100%. Tagged sequencing primers for *AR* and *MAP3K1* were designed using Primer3 version 1.1.4 (http://www.sourceforge.net) and GeneDistiller 2014 (http://www.genedistiller.org/), as follows: Forward primer (*AR*): 5′-GCATTCAGGCAGAGCTGGAT-3′; Reverse primer (*AR*): 5′-CTGCTGTGCATCAATGTGGG-3′; Forward primer (*MAP3K1*): 5′-GTTGATATATTGTCTGGTATACTGG-3′; Reverse primer (*MAP3K1*): 5′-AGGGGTCTGCAGATCATATTTTG-3′. Polymerase chain reaction (PCR) was performed in a 50-μl reaction system including 4 μl genomic DNA, 1 μl forward and reverse primers, 5 μl 10 × PCR buffer, 4 μl dNTPs, and 0.3 μl Taq Hot Start (Takara Bio, Ohtsu, Japan). The PCR conditions were as follows: an initial denaturation step (95°C for 5 min), followed by 40 cycles of denaturation (95°C for 30 s), annealing (65°C for 30 s), and elongation (72°C for 30 s). Amplicons were sequenced using an ABI 3730 system (Applied Biosystems, Foster City, Calif., U.S.A.), and sequence analysis was performed using the autoassembler software Chromas 2.6.6 (Technelysium Pty Ltd. available at www.technelysium.com.au/chromas.html and by visual inspection.

### Bioinformatic analysis

We applied Clustal W (UCD, Dublin, Ireland) software to compare 20 species to confirm the conservation of amino acids at mutated positions. To predict the potential pathogenic effects of the two variants, we used online software such as PolyPhen-2 (http://genetics.bwh.harvard.edu/pph2/), Mutation Taster (http://www.mutationtaster.org/) and PROVEAN (http://provean.jcvi.org/index.php). Modeling of wild-type and mutant proteins was performed with I-TASSER software (https://zhanglab.ccmb.med.umich.edu/I-TASSER/), and PyMOL Viewer was employed to observe the effect of the two variants on the protein.

### Site-direct mutagenesis and transient transfection

Wild-type *AR* or *MAP3K1* cDNA were synthesized and cloned into pcDNA3.1 vector by GeneArt Gene Synthesis (Thermo Fisher Scientific, Rockford, IL). *AR* variants (pcDNA3.1-*AR*-MU) and *MAP3K1* variants (pcDNA3.1-*MAP3K1*-MU) were generated by site-directed mutagenesis. To obtain the *AR* His690Glu amino acidic substitution (from cytosine to adenine at nucleotide 2070), we used the corresponding primers: primer forward ACGGGCCCTCTAGACTCGAGCGCCACCATGGAAGTGCAGTTAGGGCTGGGAAGG, primer reverse AGTCACTTAAGCTTGGTACCGACTGGGTGTGGAAATAGATGGGCTTGACTTTC. To obtain the *MAP3K1* Arg260Cys amino acidic substitution (from cytosine to thymine at nucleotide 778), the following primers were used: primer forward ACGGGCCCTCTAGACTCGAGCGCCACCATGGCGGCGGCGGCGGGGAATCGCGCC, primer reverse ACGGGCCCTCTAGACTCGAGCGCCACCATGGCGGCGGCGGCGGGGAATCGCGCC. The protocol used for site-directed mutagenesis is available upon request. All the primers were purchased from GenScript (Cayman Islands, U.K.). The presence and the construct fidelity of pcDNA3.1-*AR*-MU and pcDNA3.1-*MAP3K1*-MU were confirmed by an ABI 3730 system (Applied Biosystems, Foster City, Calif., U.S.A.).

Then, we used the high glucose Dulbecco’s Modified Eagle’s Medium containing 10% fetal bovine serum (FBS), 5% penicillin-streptomycin and 2 mM L-glutamine to culture the human embryonic kidney 293T/17 (HEK293T/17) cells (American Tissue Culture Collection, Manassas, VA). HEK293T/17 cells were transfected with plasmids containing the wild-type *AR* (pcDNA3.1-*AR*-WT) and *MAP3K1*(pcDNA3.1-*MAP3K1*-WT) or pcDNA3.1-*AR*-MU and pcDNA3.1-*MAP3K1*-MU using Lipofectamine 3000 transfection reagent (Thermo Fisher Scientific). The transfection concentration of a single plasmid was 2.5 μg/ml. When the cotransfection of the two plasmids was conducted, the concentration of each plasmid is 1.5 μg/ml.

After 48 h, cells were collected and lysed by mammalian protein extraction reagent (ThermoFisher Scientific) and protease inhibitor cocktail (Sigma Aldrich). The cells lysates were used to analyze the AR protein expression by Western blot.

### Immunoblotting

The cells lysates were mixed with Laemmli buffer containing 2-mercaptoethanol. Then, the mixtures were boiled at 95°C for 5 min. Proteins were separated by sodium dodecyl sulfate polyacrylamide gel electrophoresis (SDS-PAGE) and transferred onto nitrocellulose membranes (400 mA, 1 h). After membranes were incubated overnight with primary antibodies, they were washed two times for 10 min with 0.2% tris-buffered saline Tween. Next, membranes had been incubated with secondary antibodies for 1 h. Before 5-min incubation with chemiluminescent HRP substrate (Millipore Corporation, Billerica, U.S.A.), membranes were washed three times for 10 min with 0.2% tris-buffered saline Tween. Finally, bands were visualized by Chemidoc XRS System (Bio-Rad, Hercules, CA). The following antibodies were used: rabbit anti-AR (Abcam) and mouse anti-Actin (Servicebio).

## Results

### Clinical manifestation

**Figure 1:**
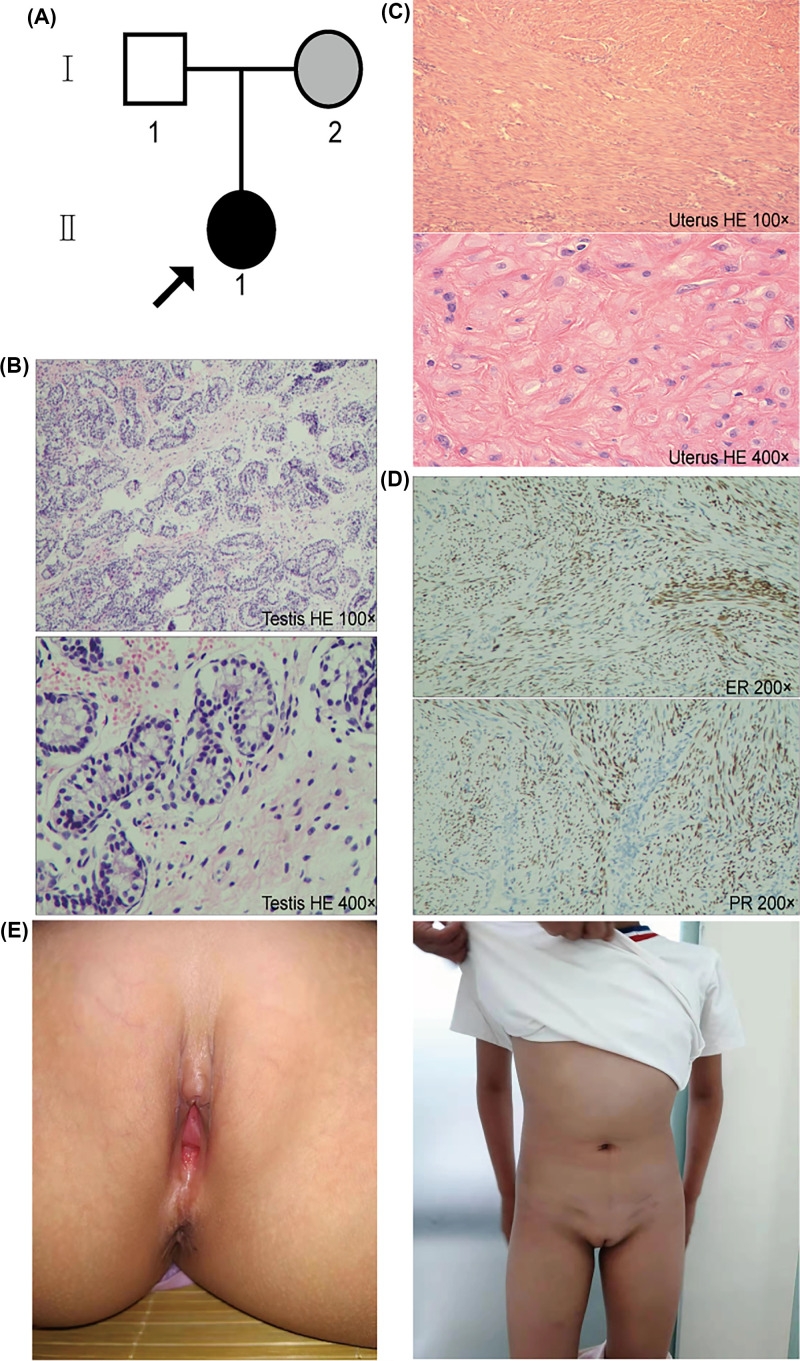
The data for the patient (**A**) The pedigree including the patient. The arrow refers to the proband. Black indicates that the person has AIS. The shading indicates that the person carries gene mutations but has a healthy phenotype. Circles indicate females reared, and squares indicate males reared. (**B**) Histopathological examination of the bilateral glands of the proband revealed bilateral underdeveloped testicular tissues. (**C**) Histopathological examination of the uterus. (**D**) Immunohistochemical staining showed positive results for estrogen and progesterone receptors in smooth muscle. (**E**) Pictures of patient II-1 at the last follow-up.

The patient’s lineage is shown in Figure 1A. Due to the discovery of bilateral inguinal hernias, the patient was admitted to the clinic at 2.6 years of age. She was born to a full-term Gravida (G)1 Pregnancy (P)1 mother. She underwent bilateral hernia repair, and a testicular mass in the left groin was unexpectedly discovered when the left hernia recurred. Karyotype analysis revealed a 46, XY karyotype. According to ultrasound examination of the pelvis, the left groin area presented a testicular echo and the right groin area a slightly stronger echo group. The uterine body echo was approximately 0.8 × 0.9 × 0.6 cm in the pelvic cavity. The posterior bladder neck exhibited a low echo, approximately 0.7 × 0.5 cm in size, and resembled a prostate echo. The boundary was clear, and the echo was homogeneous. A human chorionic gonadotropin (hCG) stimulation test was performed to determine whether she actually produced testosterone at 2.6 years of age. Consistent with our expectations, testosterone exhibited a normal rise (basal testosterone: 0.01 ng/ml; peak testosterone: 4.02 ng/ml) after hCG stimulation. Testosterone synthesis defects were excluded, and her testosterone production was consistent with the AIS phenotype. A gonadotropin-releasing hormone (GnRH) excitement test was also performed at 2.6 years of age (basal FSH: 2.28 mIU/l, peak FSH: 16.80 mIU/l; basal LH: 0.43 mIU/l, peak LH: 21.46 mIU/l), and hormone analysis indicated low testosterone, estradiol and luteinizing hormone levels for a child; however, 17α-hydroxyprogesterone, follicle-stimulating hormone, prolactin, adrenocorticotropic hormone and cortisol levels were normal for a child (Table 1). Thereafter, bilateral laparoscopic gonadectomy and bilateral gland biopsy were performed at 2.7 years of age, and histopathological examination revealed bilateral underdeveloped testicular tissues in the inguinal region (Figure 1B). At the same time, surgical exploration confirmed the presence of a uterus. Uterine histology displayed aggregates of smooth muscle (Figure 1C). Immunohistochemical staining was positive for estrogen and progesterone receptors in the smooth muscle (Figure 1D). After the operation, the patient was referred to our pediatric endocrine clinic. The patient’s height was 89.2 cm, and her body weight was 14.0 kg. Family history: The patient’s parents have remained phenotypically healthy to the present and indicated no history of familial genetic diseases or related diseases. The patient’s grandparents were deceased and could not be tested. Gynecological examination confirmed the presence of female external genitalia with no significant clitoris and labia majora, an existing vagina and the absence of pubic hair or crest.

**Table 1:** Hormone levels at the initial visit and the last follow-up

	FSH (mIU/l)	LH (mIU/l)	To (ng/ml)	E (pg/ml)	17α-OHP (ng/ml)	PRL (ng/ml)	ACTH (pg/ml)	COR (nmol/l)
Initial visit (2.6 years of age)	2.28	0.43**↓**	0.01**↓**	0.01**↓**	0.35	6.32	19.8	220.4
Last follow-up (10.3 years of age)	11.46**↑**	0.28**↓**	0.15	5.00**↓**	0.69	3.95**↓**	16.98	199.8

**Abbreviations:** 17α-OHP, 17α-hydroxyprogesterone; ACTH, adrenocorticotropic hormone; COR, cortisol; E, estradiol; FSH, follicle stimulating hormone; LH, luteinizing hormone; PRL, prolactin; To, testosterone.

**Normal reference values for the patient’s age:** FSH: 1.7–7.7 mIU/l; LH: 1.0–11.4 mIU/l; To: 0.06–0.82 ng/ml; E: 22.3–341 pg/ml; 17α-OHP: <2.32 ng/ml; PRL: 4.79–23.3 ng/ml; ACTH: 7.2–63.3 pg/ml; COR: 172–497 nmol/l.

### Follow up

The patient’s last follow-up was at 10.3 years of age, and the patient identified as female. Gynecological examination confirmed mild clitoromegaly, an existing vagina, no significant labia majora, and the absence of pubic hair or crest for the female external genitalia (Figure 1E). The patient’s mental development was normal. No breast development or menstrual cramps (Figure 1E) and no urethra or vaginal stenosis occurred. No medication was used during the follow-up period. No potential complications or developments, such as psychological or cognitive impairment, gender anxiety disorder or gonadal tumors, were observed.

Her hormone levels at the last follow-up, including testosterone, 17α-hydroxyprogesterone, follicle stimulating hormone, adrenocorticotropic hormone and cortisol, were normal for a prepubertal patient. Conversely, the patient’s estradiol, prolactin and luteinizing hormone levels were low for a prepubertal patient (Table 1).

### Variant detection

**Figure 2:**
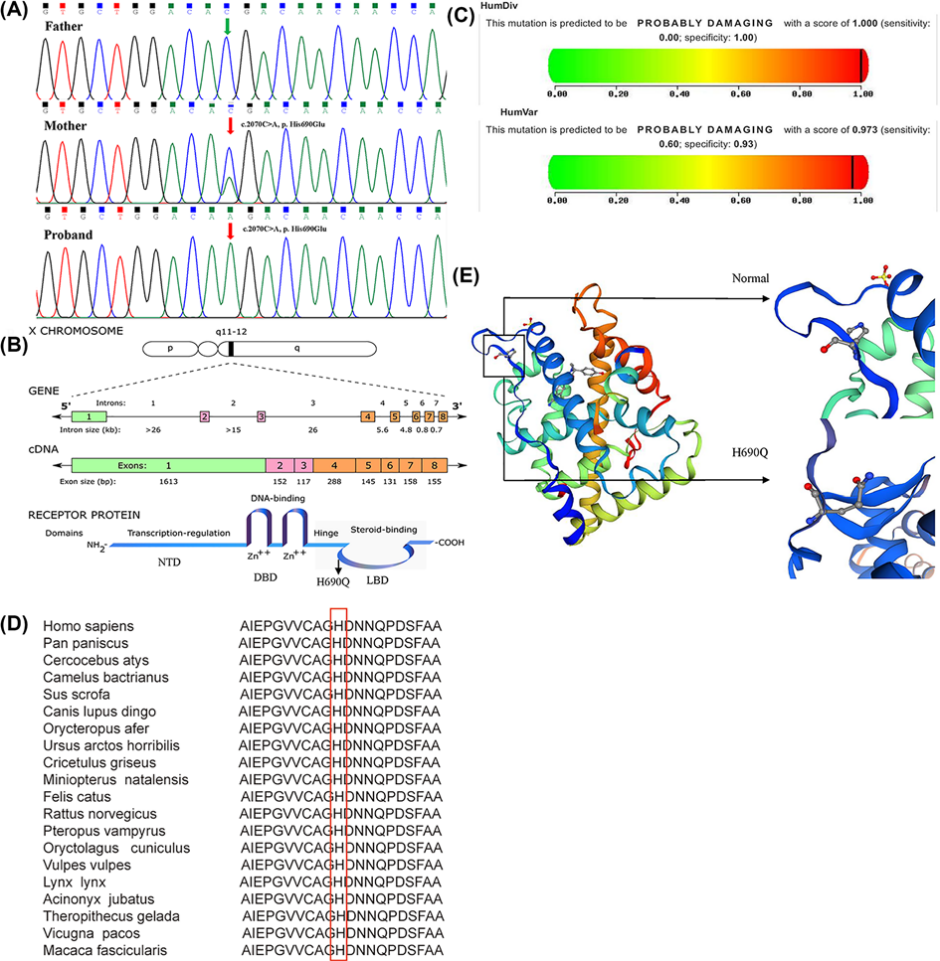
Sequencing results and bioinformatic analysis of the *AR* gene variant identified in our study (**A**) Partial sequence diagram of *AR* in our case. A novel hemizygous variant in the *AR* gene (c.2070C>A) of I-2 and II-1, resulting in the 690th amino acid of the encoded protein being mutated from histidine to glutamine, is shown by an arrow. (**B**) Location and structure of *AR*. The *AR* gene is located on chromosome Xq11-12, consists of 8 exons and encodes a protein containing 920 amino acids. Exon 1 encodes the N-terminal domain (NTD). Exons 2 and 3 encode the DNA-binding domain (DBD). Exons 3 and 4 encode the hinge region or bipartite nuclear localization, and exons 4-8 encode the ligand-binding domain (LBD). The hemizygous variant occurs at position 690 (H690Q). Original work, created from Figure 1 of “Quigley CA, De Bellis A, Marschke KB, El-Awady MK, Wilson EM, French FS. Androgen receptor defects: historical, clinical, and molecular perspectives. Endocr Rev. 1995;16:271–321”. (**C**) Score of the novel damaging variant c.2070C>A (p. His690Glu) in Polyphen v.2. (**D**) The cross-species conservation of AR around p. His690 is displayed. (**E**) Protein structure prediction of wild-type and mutant AR.

To further identify disease-causing genes to facilitate diagnosis, we subsequently applied the WES technique for genetic analysis of the pedigree. According to HGMD, we found a novel hemizygous variant in the *AR* gene (c.2070C>A) of I-2 and II-1, resulting in a change in the 690th amino acid of the encoded protein from histidine to glutamine (His690Glu, Figure 2A). The variant is absent in the GnomAD, TOPMED and ExAC databases. The androgen receptor is a nuclear ligand receptor that acts as a transcription factor. The protein consists of a central DNA-binding domain (DBD), a C-terminal ligand-binding domain (LBD) and an extended N-terminal domain (NTD), including polymorphic polyglutamine (CAG) and polyglycine (GGN) repeats. This novel variant (His690Glu) is located in the LBD and is predicted to cause an androgen-AR-binding disorder and lead to *AR* dysfunction in reproduction, growth and development (Figure 2B). Polyphen v.2 predicts the hemizygous *AR* variant to potentially be damaging, with a score of 0.973 (Figure 2C), and PROVEAN predicts this variant to be deleterious, with a score of −7.233. Mutation Taster showed that the protein properties influenced by the variant are pathogenic. The *AR* variant is “likely pathogenic” with the application of ACMG criteria. Multiple amino acid sequence alignments performed with the Clustal W tool suggested that p. His690 is conserved across various species (Figure 2D). This variant obviously causes changes in the 3D structure of the AR protein (Figure 2E).

**Figure 3:**
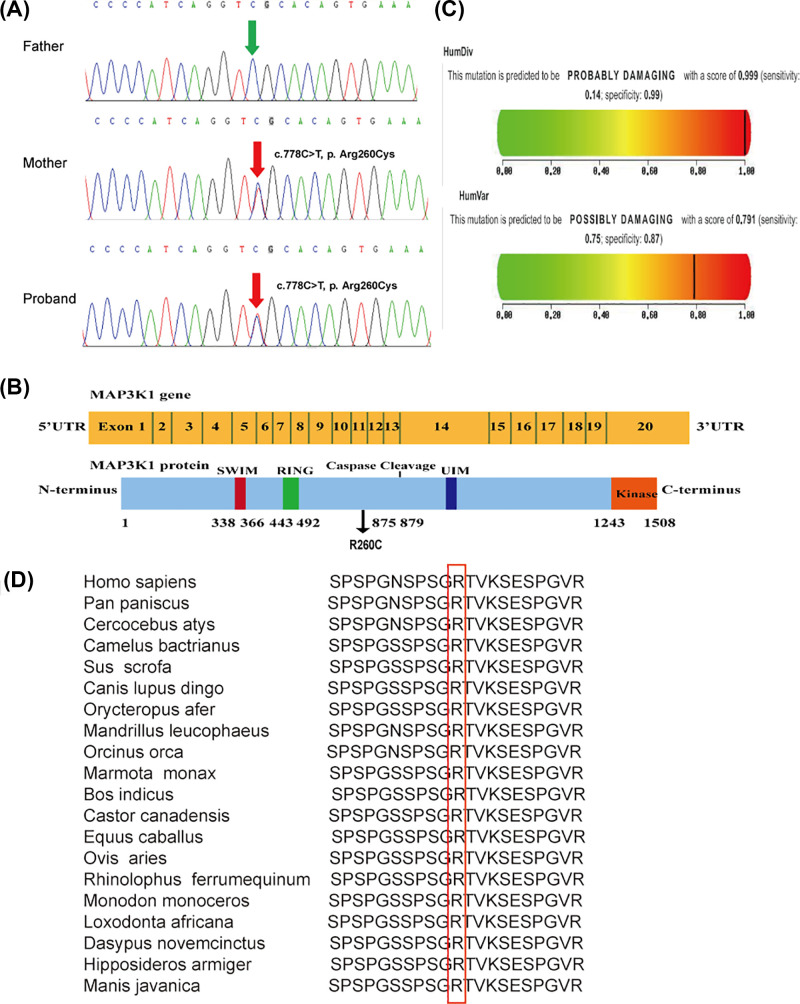
Sequencing results and bioinformatic analysis of the *MAP3K1* gene variant identified in our study (**A**) Partial sequence diagram of *MAP3K1* in our case. A rare heterozygous variant in *MAP3K1* (c.778C>T) of I-2 and II-1, resulting in the 260th amino acid of the encoded protein being mutated from arginine to cysteine, is shown by an arrow. (**B**) Structural domains of MAP3K1. Variants at the protein level are indicated below the domains. (**C**) Score of the novel damaging variant c.778C>T (p. Arg260Cys) in Polyphen v.2. (**D**) The cross-species conservation of MAP3K1 around p. Arg260 is displayed.

We also identified I-2 and II-1 as carrying a heterozygous *MAP3K1* variant (c.778C>T) that results in a change in the 260th amino acid of the encoded protein from arginine to cysteine (Arg260Cys, Figure 3A). The frequency of this *MAP3K1* variant in population databases is *T* = 0.00002 (4/248484, GnomAD_exome), *T* = 0.00001 (1/125568, TOPMED) and *T* = 0.00002 (2/113302, ExAC). The serine/threonine kinase domain is located at the C-terminus of MAP3K1, upstream of which there is a conserved caspase-3 cleavage site and a ubiquitin interaction motif (UIM). The N-terminus of MAP3K1 contains two zinc finger structures, including one RING structure and one SWIM region. The heterozygous variant occurs at position 260 (Arg260Cys) (Figure 3B). Polyphen v.2 predicts the heterozygous variant of the *MAP3K1* gene to potentially be damaging, with a score of 0.791 (Figure 3C), and PROVEAN predicts this variant to be neutral, with a score of −1.035. Mutation Taster showed that the protein properties influenced by the variant are pathogenic. The *MAP3K1* variant is a variant of “unknown significance” according to ACMG criteria. Multiple amino acid sequence alignments performed using the Clustal W tool suggested that p. Arg260 is conserved across various species (Figure 3D). However, we could not perform structural analysis because the 3D MAP3K1 protein has not yet been crystallized.

Other than the novel hemizygous *AR* variant and the rare heterozygous *MAP3K1* variant, we did not find any other DSD gene variants.

### The AR protein expression in HEK293T/17 cells

**Figure 4:**
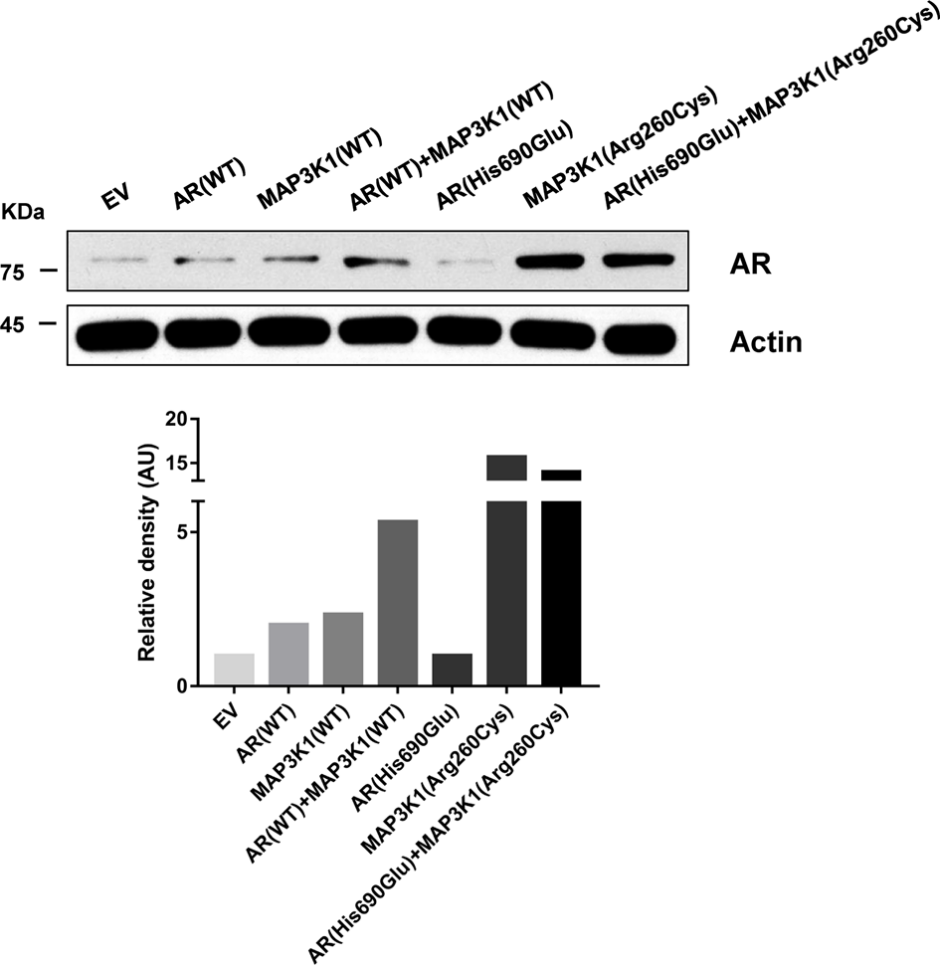
Western blotting analysis of AR production in HEK 293T/17 cells transfected with wild-type *AR* and *MAP3K1* or mutant *AR* and *MAP3K1* plasmid The AR production of HEK293T/17 cells transfected with the pcDNA3.1-*AR*-MU decreased significantly by approximately 50% compared with that of cells transfected with pcDNA3.1-*AR*-WT. Cells transfected with both wild-type plasmids showed a significantly increase in AR protein production, compared to cells with pcDNA3.1-*AR*-WT or pcDNA3.1- *MAP3K1*-WT (2.67 times; 2.29 times, respectively). This result indicates that pcDNA3.1- *MAP3K1*-WT can increase the expression of AR. The AR production of HEK293T/17 cells transfected with pcDNA3.1- *MAP3K1*-MU was 6.73 times higher than that of cells with pcDNA3.1- *MAP3K1*-WT transfection, indicating that the effect of increasing AR production became stronger after *MAP3K1* was mutated. Compared to cells transfected with the pcDNA3.1-*AR*-MU, cells transfected with both mutant plasmids showed a significantly increase in AR protein production (approximately 14 times), which also indicated that the effect of increasing AR production became stronger after *MAP3K1* was mutated. AR, androgen receptor; EV: empty vector; MAP3K1, mitogen-activated protein kinase kinase kinase 1; WT: wild-type.

To determine the effect of the *AR* and *MAP3K1* variants at the protein level, plasmids containing the wild-type *AR* and *MAP3K1* cDNA or carrying *AR* and *MAP3K1* variants were transfected into HEK293T/17 cells. The AR production of HEK293T/17 cells transfected with the pcDNA3.1-*AR*-MU decreased significantly by about 50% compared with that of cells transfected with pcDNA3.1-*AR*-WT. Cells transfected with both wild-type plasmids showed a significantly increase in AR protein production, compared to cells with pcDNA3.1-*AR*-WT or pcDNA3.1- *MAP3K1*-WT (2.67 times; 2.29 times, respectively). This result indicates that pcDNA3.1- *MAP3K1*-WT can increase the expression of AR. The AR production of HEK293T/17 cells transfected with pcDNA3.1- *MAP3K1*-MU was 6.73 times higher than that of cells with pcDNA3.1- *MAP3K1*-WT transfection, indicating that the effect of increasing AR production became stronger after *MAP3K1* was mutated. Compared to cells transfected with the pcDNA3.1-*AR*-MU, cells transfected with both mutant plasmids showed a significantly increase in AR protein production (approximately 14 times), which also indicated that the effect of increasing AR production became stronger after *MAP3K1* was mutated (Figure 4).

### Prediction of the relationship between *AR* and *MAP3K1*

**Figure 5:**
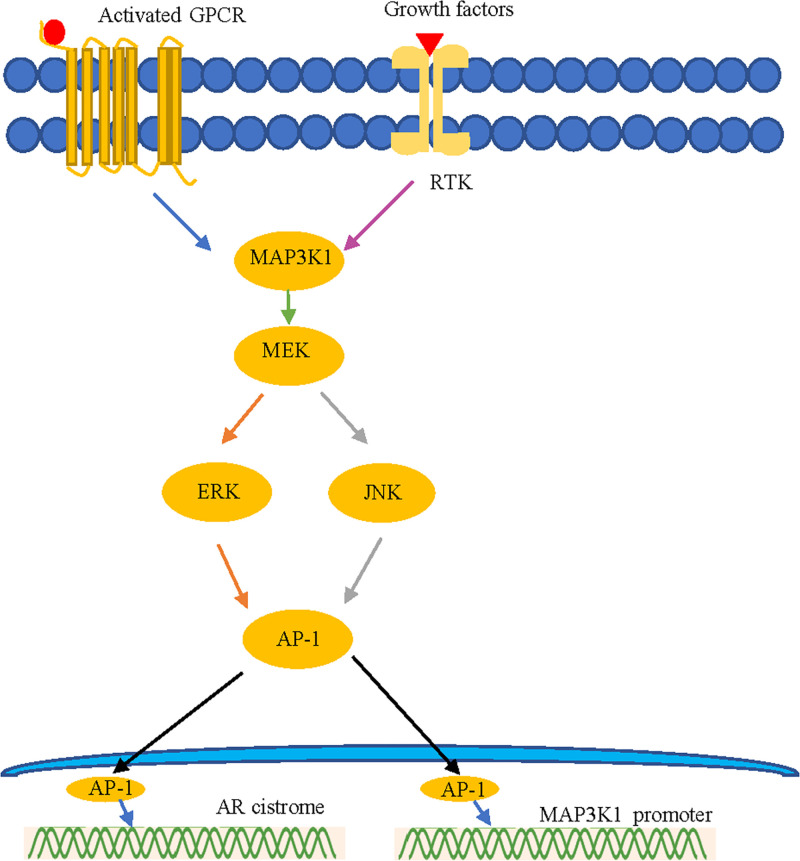
Prediction of the relationship between *AR* and *MAP3K1* Abbreviations: ERK, extracellular signal-regulated kinase; GPCR, G protein-coupled receptor; JNK, c-Jun NH2-terminal kinase; RTK, receptor tyrosine kinase.

MAP3K1 is expected to activate the transcription factor AP-1 [16,17], which may play an important role in the *AR* cistrome [18], leading to the AIS phenotype and genetic heterogeneity. In addition to studies showing that the kinase activity of MAP3K1 is required for the maximum activity of AP-1 [19], other studies have shown that AP-1 may be involved in regulation of the *MAP3K1* promoter [20]. Casalino et al. demonstrated that MEK/ERK-mediated AP-1 phosphorylation dramatically increases the AP-1 half-life [21]. Therefore, the observed heterogeneity may be caused by the MAP3K1–MEK–ERK–AP-1–AR axis. Furthermore, activation of *MAP3K1* induces JNK and downstream signaling pathways [22], and the use of chemical inhibitors to inhibit JNK leads to significant inhibition of *AR* expression [23]. These results suggest that the MAP3K1–JNK–AR axis may play an important role in the phenotype and genotype heterogeneity of the patient examined in this study (Figure 5).

## Discussion

In the present study, we describe a patient with AIS with a novel hemizygous variant in the *AR* gene (c.2070C>A, p. His690Glu) and a rare heterozygous variant in the *MAP3K1* gene (c.778C>T, p. Arg260Cys). Bioinformatics analysis predicted these two variants to be pathogenic. And we characterized at a molecular level the pathogenic variants in the *AR* and *MAP3K1* genes that affect the production of AR protein. The rare heterozygous variant in the *MAP3K1* gene might act as a genetic modifier of the phenotype, as the patient has rudimentary Müllerian structures, which is uncommon in AIS. Therefore, we consider that the two pathogenic variants are the cause of AIS and that they are the exclusive causative agents of the phenotype of the described patient. Moreover, the confirmation of an AIS diagnosis by WES can reveal additional genetic variants to explain the heterogeneity of the disease.

AIS is highly heterogeneous and divided into three categories according to the degree of genital masculinization: mild androgen insensitivity syndrome (MAIS), partial androgen insensitivity syndrome (PAIS) and complete androgen insensitivity syndrome (CAIS). The clinical phenotypes range from a typical male habitus with mild spermatogenic defects and reduced secondary terminal hair to a full female habitus despite the presence of a Y chromosome. The correlation of genotype and phenotype has not yet been established.

AIS is a congenital disorder in which a defect in the *AR* gene leads to cellular resistance to androgens. More than 1000 AIS-causing mutations in the *AR* gene have been identified [24]. The types of *AR* gene variants include gene deletions, splice site mutations, premature stop codons and missense mutations, among others. The most common genetic abnormalities are missense mutations, which often occur in two important segments of the receptor protein: the DBD and LDB regions. Missense mutations that result in a single amino acid substitution are known to produce the most phenotypic diversity.

**Figure 6:**
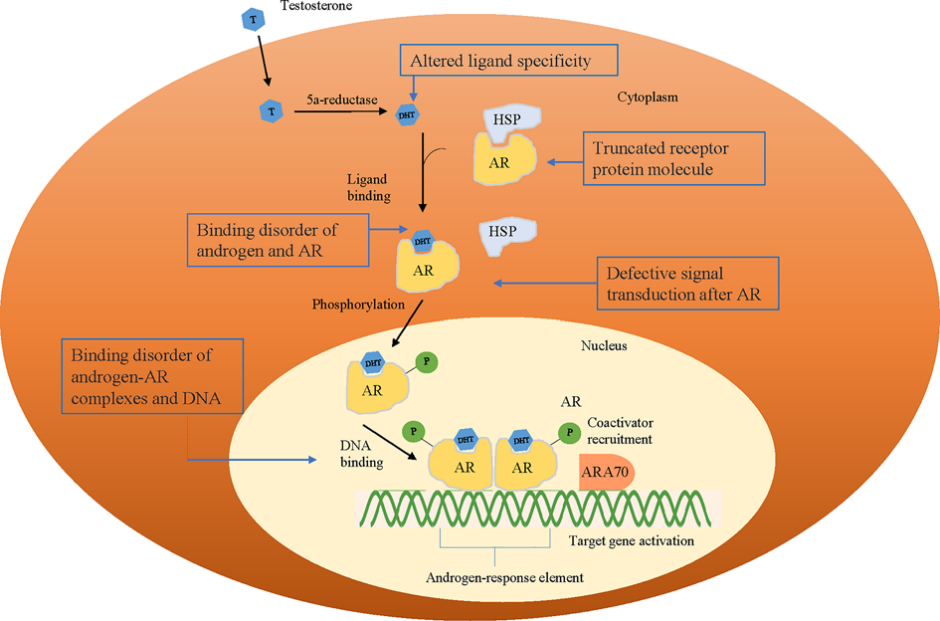
Five potential mechanisms by which *AR* variants reduce or abolish the function of *AR* Abbreviations: AR, androgen receptor; ARA70, androgen receptor coactivator 70; DHT, dihydrotestosterone; HSP, heat shock protein.

There are at least five potential mechanisms by which *AR* variants reduce or abolish AR function (Figure 6): androgen and AR binding disorders, androgen–AR complex and DNA-binding disorders, truncated AR proteins, altered ligand specificity and defective signal transduction downstream of AR. Impairment in androgen or DNA binding is the most common mechanism [25,26]. First, exons 5–8 and part of exon 4 encode the LBD, which includes residues 664–920 [27], and variants that cause androgen–AR-binding disorders are commonly reported in exons 4–8 [28,29–31]. Patients can present complete feminization of the testes or incomplete testicular feminization [26,29,31,32]. Second, exons 2 and 3 encode the DBD, from residue 559 to 624 [27], and variants that cause binding disorders of androgen–AR complexes and DNA mainly occur in these two exons. Patients exhibit complete testicular feminization or incomplete testicular feminization, possibly accompanied by the development of cancer [33–36]. Third, the incidence of variants causing truncation of the receptor protein molecule is low. Patients often have the classic clinical/endocrine phenotype of CAIS [37–40]. Fourth, variants that cause ligand-specific changes are rare. Such variants alter the binding properties of AR with androgens, such as Thr877Ala [41]. A mutant AR exhibits a poor binding affinity to testosterone or dihydrotestosterone but a high binding affinity to progesterone and other steroid hormones. Fifth, under normal conditions, interaction occurs between the N-terminus and the C-terminus of AR, with a regulatory effect on signal transduction downstream of AR. Some variants (such as Met742Val, Phe725Leu, Gly743Val, Phe754Leu and Met886Val) [42] result in abnormal interaction between the AR N- and C-termini, which results in AR dysfunction. However, AR function can be restored via induction by high-dose androgen.

**Figure 7:**
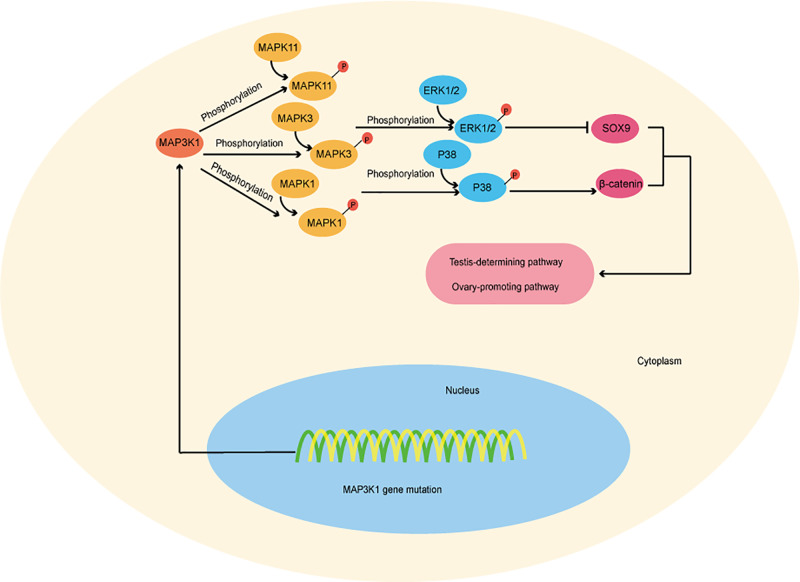
Diagram of the *MAP3K1* gene-associated mechanism resulting in 46, XY DSD The *MAP3K1* gene mutation causes increased phosphorylation of the downstream MAP kinase pathway targets–MAPK11, MAP3K and MAPK1, thereby promoting phosphorylation of ERR1/2 and p38 and ultimately leading to 46, XY DSD. Increased phosphorylation of ERK1/2 and p38 may result in decreased expression of SOX9 and increased activity of β-catenin, respectively, which are important signaling molecules in testicular and ovarian-promoting pathways.

In addition to the *AR* gene, many other genes have variants that cause disorders of sex development, such as the *MAP3K1* gene. *MAP3K1* is part of the gene network responsible for gonadal development. *MAP3K1* is involved in regulating the transcription of many important genes, including c-Jun and c-Fos, and plays an important role in cell proliferation, differentiation and apoptosis [15]. The serine/threonine kinase domain is located at the C-terminus of MAP3K1, upstream of which there is a conserved caspase-3 degradation site (the homologous gene in humans is *DTDDG*) and a ubiquitin interaction motif (UIM). As mentioned above, two zinc finger structures, including one RING structure and one SWIM region, are present at the N-terminus of MAP3K1 [43]. The cleavage and isolation of a C-terminal kinase by caspase-3 are required for MAP3K1 to induce apoptosis, and the role of UIM is unclear. The SWIM domain is an ancient domain found in the bacterial SWI2/SNF2 ATPase and plant MuDR transposase and directly binds to c-Jun, which is required for MAP3K1-mediated c-Jun ubiquitination. The RING domain shows a typical pattern of plant homology domains. Similar to many RING-containing proteins, MAP3K1 exhibits E3 ubiquitin ligase activity. MAP3K1 ubiquitination requires functional RING and kinase domains [44–46]. Studies have also shown that *MAP3K1* gene variants result in altered binding of cofactors and increase phosphorylation of the downstream MAP kinase pathway targets MAPK11, MAP3K and MAPK1, thereby promoting ERR1/2 and p38 phosphorylation and ultimately leading to 46, XY DSD. Increased phosphorylation of ERK1/2 and p38 may result in decreased expression of SOX9 and increased activity of β-catenin, respectively, which are important signaling molecules in testicular and ovarian-promoting pathways (Figure 7) [47–49].

Interestingly, we detected a compound variant of the *MAP3K1* and *AR* genes in a 46, XY Chinese patient with AIS. This patient exhibited a uterus in the pelvic region, which was indicative of incomplete Müllerian regression and did not match the typical characteristics of AIS. In individuals with AIS, Müllerian derivatives are generally not found; indeed, only six articles have reported the presence of a uterus in a patient with AIS [12,50–54]. This situation prompted us to consider why the patient developed this phenotype. Unlike the six studies mentioned above, in which deleterious variants were only detected in the *AR* gene, this is the first study to perform WES to detect a novel hemizygous variant of *AR* (c.2070C>A, p. His690Glu) and a rare heterozygous variant of *MAP3K1* (c.778C>T, p. Arg260Cys). A missense mutation in the *AR* gene (c.2069A>C, p. His690Pro) has been reported in the same codon [55]. Rosa et al. reported a 16-year-old girl with all of the characteristics of CAIS, i.e. primary amenorrhea, no axillary or pubic hair, female external genitalia, no uterus and abdominal testes. The karyotype was 46, XY. The gonads were located in the abdominal cavity, and histological examination of the gonads revealed testicular tissue. After hCG stimulation, testosterone showed a normal rise, and testosterone synthesis defects were thus excluded (Table 2). Here, we describe a 2.6-year-old girl with AIS. Our patient manifested bilateral inguinal masses (pathologically confirmed as bilateral testicular tissue), female external genitalia and the presence of a uterus, which was indicative of incomplete Müllerian regression and did not match the typical characteristics of AIS. After hCG stimulation, testosterone also displayed a normal rise, excluding testosterone synthesis defects. These results hint that the rare variant in the *MAP3K1* gene might be related to the clinical heterogeneity of AIS.

**Table 2:** Phenotype comparison of patients with a change at p. His690 of AR

Protein change	p. His690Pro	p. His690Glu
Gene change	c.2069A>C	c.2070C>A
Codon change	CAC-CCC	CAC-CAA
Age (years)	16.0	2.6
External genitalia	Female external genitalia	Female external genitalia
Gonadal location	Abdomen	Bilateral inguinal region
Histopathological examination	Testicular tissue	Testicular tissue
Müllerian structures	None	Uterus
Axillary and Pubic hair	None	None
Breast development	Normal	Not
Menses	Primary amenorrhea	Not
T resp. hCG	Elevated	Elevated
Novel or known	Rosa et al.,2002	Novel

To explain the molecular mechanism underlying our genetic findings, we carried out *in vitro* experiments of the *AR* and *MAP3K1* variants. His690Glu is an *AR* mutation that decreased the AR production, whereas the Arg260Cys mutation of *MAP3K1* increased the AR production. The novel compound variants of the *AR* and *MAP3K1* genes also increased the production of AR protein. Taken together, these findings suggest that the *AR* and *MAP3K1* variants severely affect the AR protein production, explaining the uncommon phenotype in the patient carrying these variants. Furthermore, we predicted the relationship between *AR* and *MAP3K1*. MAP3K1 is expected to activate the transcription factor AP-1 [16], which may play an important role in the *AR* cistrome [18], leading to an AIS phenotype and genetic heterogeneity. In addition to reports showing that the kinase activity of MAP3K1 is required for the maximum activity of AP-1 [19], other studies have shown that AP-1 might be involved in *MAP3K1* promoter regulation [20]. Casalino et al. demonstrated that MEK/ERK-mediated AP-1 phosphorylation dramatically increases the AP-1 half-life [21]. Overall, this heterogeneity may be caused by defects in the MAP3K1–MEK–ERK–AP-1–AR axis. In addition, activation of MAP3K1 leads to activation of JNK and downstream signaling pathways [22]. Furthermore, chemical inhibitors of JNK significantly suppress AR expression [23]. These results suggest that the MAP3K1–JNK–AR axis may play an important role in the phenotype and genotype heterogeneity of the patient described in the present study.

It is undeniable that the present study has some limitations. The study included a 46, XY Chinese female patient with AIS whose grandparents had died, and we were unable to trace potential genetic variation. In addition, there is a lack of basic experiments to validate regulatory mechanism between the *AR* and *MAP3K1* gene. Our findings extend the gene variant profile of *AR* and *MAP3K1*. However, the association between the phenotype and genotype of AIS remains unclear, and more patients need to be studied to provide more comprehensive data.

In summary, our study contributes to the known heterogeneity of AIS, which has significance for research on the heterogeneity of diseases. The widespread use of WES may reveal further genetic variants, and some genetic variants may help to explain genotype–phenotype associations.

## Abbreviations

ACMG: American College of Medical Genetics and Genomics
AIS: androgen insensitivity syndrome
AR: androgen receptor
DBD: DNA-binding domain
DSD: disorders of sex development
ERK: extracellular signal-regulated kinase
ExAC: the Exome Aggregation Consortium
GnomAD: Genome Genome Aggregation Database
GnRH: Gonadotropin-releasing hormone
hCG: human chorionic gonadotropin
HGMD: Human Gene Mutation Database
HGVS: Human Genome Variation Society
JNK: c-Jun NH2-terminal kinase
LBD: ligand-binding domain
NTD: N-terminal domain
OMIM: Online Mendelian Inheritance in Man
PCR: polymerase chain reaction
TOPMED: Trans-Omics for Precision Medicine
UIM: ubiquitin interaction motif
WES: whole exome sequencing
